# Prognostic value of a computer-aided diagnosis system involving bone scans among men treated with docetaxel for metastatic castration-resistant prostate cancer

**DOI:** 10.1186/s12885-016-2160-1

**Published:** 2016-02-16

**Authors:** Koichi Uemura, Yasuhide Miyoshi, Takashi Kawahara, Shuko Yoneyama, Yusuke Hattori, Jun-ichi Teranishi, Keiichi Kondo, Masatoshi Moriyama, Shigeo Takebayashi, Yumiko Yokomizo, Masahiro Yao, Hiroji Uemura, Kazumi Noguchi

**Affiliations:** Department of Urology and Renal Transplantation, Yokohama City University Medical Center, 4-57 Urafune-cho, Minami-ku, Yokohama, 232-0024 Japan; Department of Urology, Yokohama Municipal Citizen’s Hospital, Yokohama, Japan; Department of Radiology, Yokohama City University Medical Center, Yokohama, Japan; Department of Urology, Yokohama City University Graduate School of Medicine, Yokohama, Japan

**Keywords:** Prostate cancer, Castration-resistant, Survival prediction, Bone scan index

## Abstract

**Background:**

The bone scan index (BSI), which is obtained using a computer-aided bone scan evaluation system, is anticipated to become an objective and quantitative clinical tool for evaluating bone metastases in prostate cancer. Here, we assessed the usefulness of the BSI as a prognostic factor in patients with metastatic castration-resistant prostate cancer (mCRPC) treated using docetaxel.

**Methods:**

We analyzed 41 patients who received docetaxel for mCRPC. The Bonenavi system was used as the calculation program for the BSI. The utility of the BSI as a predictor of overall survival (OS) after docetaxel was evaluated. The Cox proportional hazards model was used to investigate the association between clinical variables obtained at docetaxel treatment, namely PSA, patient age, liver metastasis, local therapy, hemoglobin (Hb), lactase dehydrogenase (LDH), albumin (Alb), PSA doubling time, and BSI and OS.

**Results:**

The median OS after docetaxel therapy was 17.7 months. Death occurred in 22 (53.7 %) patients; all deaths were caused by prostate cancer. In multivariate analysis, three factors were identified as significant independent prognostic biomarkers for OS after docetaxel; these were liver metastases (yes vs no; HR, 3.681; *p* = 0.026), Alb (<3.9 vs ≥3.9; HR, 3.776; *p* = 0.020), and BSI (>1 % vs ≤1 %; HR, 3.356; *p* = 0.037). We evaluated the discriminatory ability of our models including or excluding the BSI by quantifying the c-index. The BSI improved the c-index from 0.758 to 0.769 for OS after docetaxel. CRPC patients with a BSI >1 had a significantly shorter OS than patients with a BSI ≤1 (*p* = 0.029).

**Conclusions:**

The BSI, liver metastases and Alb were independent prognostic factors for OS after docetaxel. The BSI might be a useful tool for risk stratification of mCRPC patients undergoing docetaxel treatment.

## Background

Huggins and Hodges [1] reported the efficacy of androgen deprivation therapy for advanced prostate cancer in 1941. Although 80–90 % of prostate cancers with metastasis respond to initial androgen ablation therapy, most patients finally develop metastatic castration-resistant prostate cancer (mCRPC) [2, 3]. Patients with mCRPC exhibit progression of systemic symptoms and local complications.

Docetaxel is a survival-prolonging chemotherapy drug used to treat mCRPC patients. Two clinical trials, TAX327 and SWOG99-16, have reported a 20–30 % relative improvement in overall survival (OS) relative to mitoxantrone, with a median improvement of 2–3 months [4–6].

There have been many reports from Asia and Western countries regarding prognostic factors and risk classification in relation to docetaxel treatment [7–20]. In a previous study, we reported risk factors for mCRPC patients before docetaxel treatment [18]. In results, pain, visceral metastasis, anemia and progression of bone metastasis were found to be independent prognostic factors for OS after docetaxel. In relation to these prognostic factors “bone metastasis progression” is a subjective variable; thus, an objective and quantitative scoring system for bone metastasis evaluation would potentially be a strong prognostic factor. Recently, a computer-aided diagnosis system (Bonenavi) for bone scans has been developed. This system can be used to calculate the bone scan index (BSI); this provides an objective and quantitative measure of the percentage of the skeleton affected by bone metastases [21]. Mitsui et al. found that patients with a decreased BSI after taxane-based chemotherapy had significantly longer OS than other patients [22]. They also reported that patients with a BSI ≥3.0 % had reduced survival relative to men with a BSI <3.0 % in the same cohort. In our study, we analyzed the relationship between mCRPC prognosis and clinicopathological factors, including the BSI calculated using the Bonenavi system for OS after docetaxel. The aim of our study was to determine if BSI could be a useful prognostic marker for mCRPC patients undergoing docetaxel treatment.

## Methods

### Study design, patients and treatments

We retrospectively analyzed 41 patients who were treated with docetaxel for mCRPC between 2011 and 2014 at Yokohama City University Medical Center and Yokohama University Hospital. Patient characteristics are listed in Table 1. Of the patients, 22 had only bone metastasis, 8 had lymph node + bone metastasis, 2 had lung + lymph node + bone metastasis, 3 had lung + bone metastasis, and 6 had liver + lymph node + bone metastasis. The initial median PSA value was 176.0 ng/ml and the PSA level at docetaxel induction was 56.8 ng/mL. The median BSI was 2.4 % (range, 0.0–12.2 %). All patients had histologically confirmed prostate adenocarcinoma. The 2009 TNM clinical staging system and 2005 International Society of Urologic Pathology Gleason grading system were used [23]. In all patients, clinical stage was evaluated using chest and body computed tomography and bone scans at initial treatment, and before docetaxel treatment.

**Table 1:** Patient characteristics (*n* = 41)

Variables before primary androgen deprivation therapy	
Median initial PSA, ng/mL (range)	176.0 (4.2-6500.0)
Gleason scores, *n* (%)	
6-7	4 (9.8 %)
8-10	37 (90.2 %)
Clinical T, *n* (%)	
≤T3	32 (78.0 %)
T4	9 (22.0 %)
Clinical N, *n* (%)	
N0	14 (34.1 %)
N1	27 (65.9 %)
Clinical M, *n* (%)	
M0	12 (29.3 %)
M1a	5 (12.2 %)
M1b	21 (51.2 %)
M1c	3 (7.3 %)
EOD, *n* (%)	
0	17 (41.5 %)
1	4 (9.8 %)
2	4 (9.8 %)
3	7 (17.1 %)
4	9 (22.0 %)
Variables at docetaxel therapy induction	
Median PSA, ng/mL (range)	56.8 (0.2-4715.0)
Median PSA doubling time, months (range)	1.4 (−3.8-47.1)
Median LDH, IU/L (range)	225 (135–1129)
Median Hb, g/dL (range)	11.7 (8.1-13.7)
Median Alb, g/dL (range)	3.9 (2.4-4.7)
Median ALP, IU/L (range)	278 (135–3296)
Median age, years (range)	73 (48–81)
EOD, *n* (%)	
0	1 (2.4)
1	10 (24.4)
2	13 (31.7)
3	11 (26.8)
4	6 (14.6)
Median BSI, % (range)	2.4 (0.0-12.2)
BSI ≤1 %, *n* (%)	18 (43.9 %)
BSI >1 %, *n* (%)	23 (56.1 %)
Liver metastasis, *n* (%)	
Yes	6 (14.96 %)
No	35 (85.4 %)
Local therapy (prostatectomy or radiation), *n* (%)	
Yes	5 (12.2 %)
No	36 (87.8 %)

*PSA* prostate-specific antigen, *LDH* lactate dehydrogenase, *Hb* hemoglobin, *Alb* albumin, *BSI* bone scan index

Each hospital used the same treatment protocol. All patients were initially treated with androgen deprivation therapy (medical or surgical castration with anti-androgen). After initial androgen ablation therapy failed, almost all patients were given anti-androgen withdrawal therapy, subsequent substitution treatment comprising anti-androgen therapy (bicalutamide to flutamide), followed by docetaxel with dexamethasone. Patients were treated with 60 mg/m^2^ docetaxel IV on day 1, given over 1 h, every 21 days. All patients received continuous androgen ablation therapy (an LH-RH analogue) and 0.5 mg dexamethasone orally. Treatment was continued until disease progression or unacceptable adverse events occurred.

Some patients received bisphosphonate or denosumab after the development of CRPC. No patients received abiraterone, enzalutamide or cabazitaxel because these drugs were not approved until 2013. For patients at the terminal stage, palliative therapy and pain control with morphine, palliative external beam radiotherapy, and strontium were used as appropriate. Serum PSA levels were measured using the Elecsys PSA Assay (Roche Diagnostics Corp., Basle, Switzerland).

### BSI

Bone scan images were obtained within 1 month before or after docetaxel treatment induction. The automated method for analysis of anterior and posterior whole-body bone scan images has been described previously [24]. Each individual hot spot was classified as a metastasis or not a metastasis using an artificial neural network (ANN), and the BSI was calculated as the percentage of the sum of all hot spots classified as bone metastases using the ANN values. To calculate the BSI, we used Bonenavi version 2 (Fujifilm RI Pharma Co. Ltd., Tokyo, Japan; Exini Bone, Exini Diagnostics, Lund, Sweden) [25].

### Statistical analysis

We compared ALP or EOD and BSI levels using Pearson’s correlation coefficient. If some factors were found to be correlated with other factors, the correlated factors were not analyzed simultaneously because of multicollinearity. We investigated the usefulness of a BSI as a predictor of survival after docetaxel treatment. The Cox proportional hazards model with stepwise regression analysis was used to investigate the association between clinical variables obtained at docetaxel treatment namely PSA, patient age, liver metastasis, local therapy, hemoglobin (Hb), lactase dehydrogenase (LDH), albumin (Alb), PSA doubling time, BSI and survival. The cutoff point for PSA, patient age, Hb, LDH, Alb and PSADT were determined using the median value of each variable. The cutoff point for the BSI was set at 1.0 % according to a report by Ulmert and colleagues [24].

Relative risks and 95 % confidence intervals were derived. The C-index was used regarding the discriminatory ability of our models.

To calculate the PSA doubling time (PSADT), two consecutive PSA values, spaced 1 month apart after docetaxel induction therapy, were used. PSADT was calculated using the natural log of 2 (0.693) divided by the slope of the relationship between the log of PSA and the time of PSA measurement for each patient [26].

The Kaplan–Meier product-limit estimator was used to estimate the survival distribution. The log-rank test was used for analysis of the survival differences.

All tests were two-sided, and the significance level was fixed at alpha = 0.05. All analyses were conducted using IBM SPSS Statistics software for Windows, version 22 (IBM Corp., Armonk, NY, USA) and the R stats package (R Foundation for Statistical Computing, Vienna, Austria).

The experimental procedures were conducted in accordance with the ethical standards of the Helsinki Declaration. This study was approved by the institutional review board of Yokohama City University Medical Center. Informed consent to participate in the study were obtained from participants

## Results

**Fig. 1:**
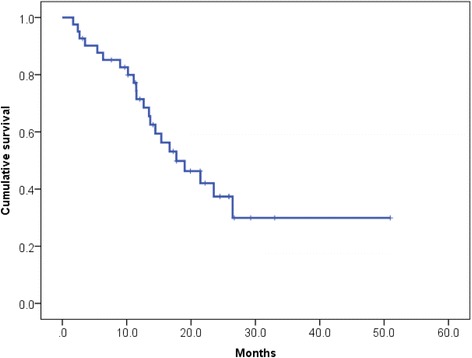
Kaplan–Meier curve for overall survival (OS) after docetaxel induction therapy. The median OS after docetaxel was 17.7 months

In our cohort, death occurred in 22 patients (53.7 %); all deaths were caused by prostate cancer. The median OS after docetaxel therapy was 17.7 (95 % confidential interval [CI]: 10.6–24.9; range, 1.7–51.0) months (Fig. 1).

**Fig. 2:**
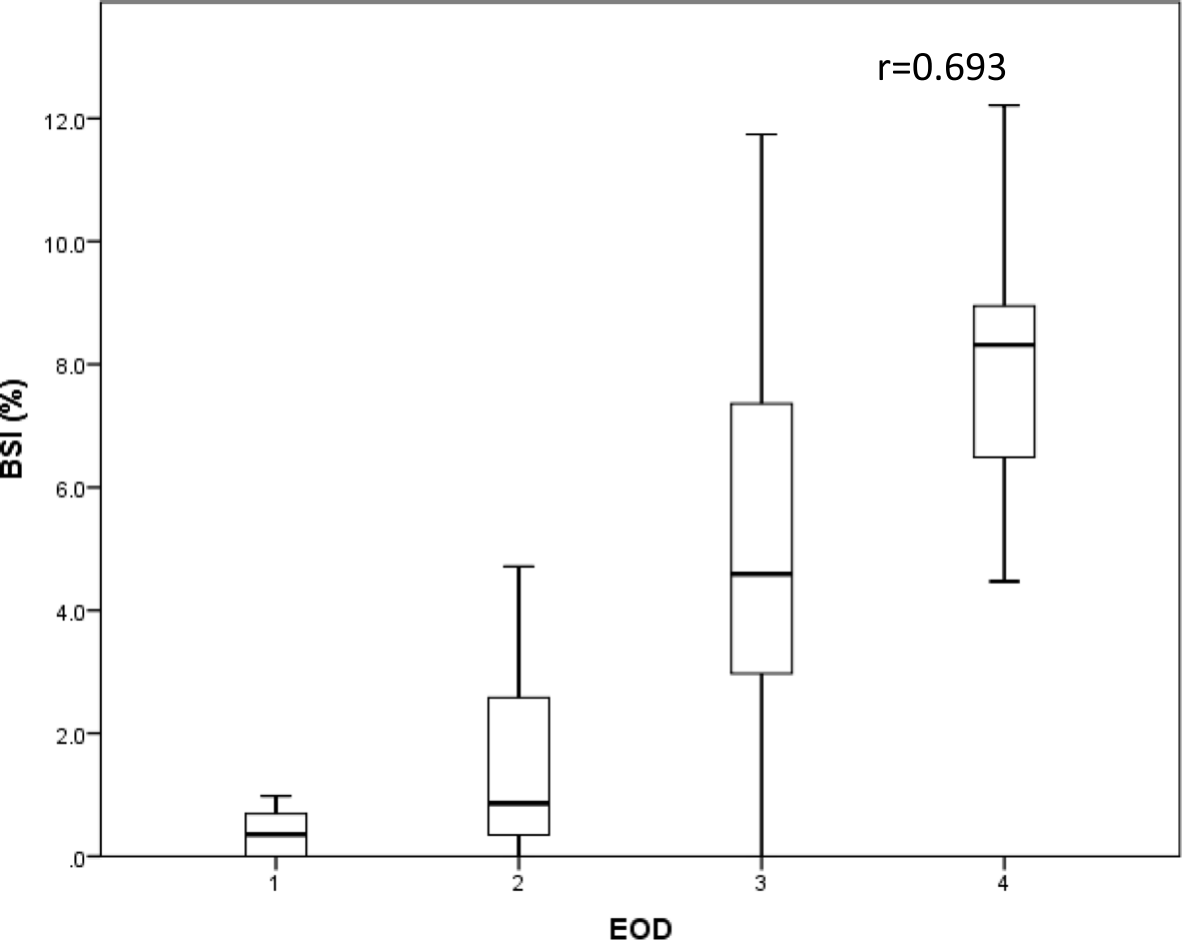
Correlation of EOD classification and the BSI. Box plots indicate the first and third quartiles. The band inside the box shows the median. Lines extending vertically from the boxes (whiskers) indicate variability outside the upper and lower quartiles. There was a significant correlation between EOD and the BSI (*r* = 0.693). BSI; bone scan index, EOD; extent of disease on bone scan

**Fig. 3:**
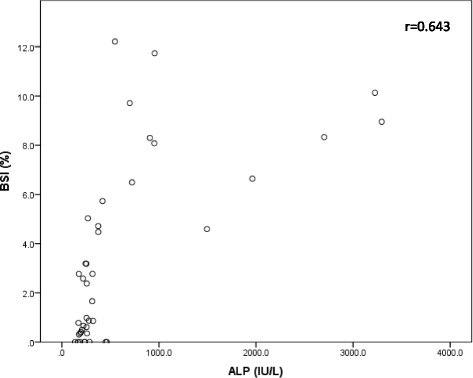
Correlation of ALP levels and the BSI. There was a strong correlation between serum ALP levels and the BSI (*r* = 0.643). ALP; alkaline phosphatase, BSI; bone scan index

The correlation of EOD classification and the BSI is shown in Fig. 2. There was a significant correlation between EOD and the BSI (*r* = 0.693). A significant correlation between serum alkaline phosphatase (ALP) levels and the BSI was also observed (r = 0.643, Fig. 3). Because of the strong correlations, we did not analyze the BSI, serum ALP levels, and EOD in multivariate analysis simultaneously owing to multicollinearity.

In multivariate analysis, three factors were identified as independent prognostic biomarkers for OS after docetaxel therapy as follows: liver metastases (yes vs no; HR, 3.681; 95 % CI, 1.166–11.616; *p* = 0.026), Alb (<3.9 g/dL vs ≥3.9 g/dL; HR, 3.776; 95 % CI, 1.238–11.516; *p* = 0.020), and BSI (>1 % vs ≤1 %; HR, 3.356; 95 % CI, 1.078–10.453; *p* = 0.037) (Table 2).

**Table 2:** Univariate and multivariate analysis of overall survival after the administration of docetaxel

Variables at docetaxel induction	Univariate analysis				Multivariate analysis			
	*p* value	Hazard ratio	95.0 % CI		*p* value	Hazard ratio	95.0 % CI	
			Lower	Upper			Lower	Upper
PSA (≥56.8 ng/mL vs <56.8)	0.115	1.991	0.845	4.693	0.894	0.929	0.317	2.729
Age(>73 vs ≤73)	0.623	0.810	0.349	1.879	0.944	0.964	0.343	2.704
Liver mets (yes vs no)	0.239	1.825	0.670	4.969	0.026	3.681	1.166	11.616
Local therapy (yes vs no)	0.137	0.218	0.029	1.624	0.206	0.213	0.019	2.339
Hb (<11.7 g/dL vs ≥11.7)	0.072	0.457	0.194	1.072	0.887	1.073	0.404	2.850
LDH (>225 IU/L vs ≤225)	0.045	2.385	1.019	5.578	0.078	2.500	0.903	6.915
Alb (<3.9 g/dL vs ≥3.9)	0.001	5.965	1.987	17.908	0.020	3.776	1.238	11.516
PSADT (≤1.4 months vs >1.4)	0.123	1.971	0.832	4.668	0.888	1.075	0.392	2.948
BSI (>1 % vs ≤1 %)	0.035	2.673	1.072	6.661	0.037	3.356	1.078	10.453

*PSA* prostate-specific antigen, *PSADT* PSA doubling time, *Hb* hemoglobin, *LDH* lactate dehydrogenase, *Alb* albumin, *BSI* bone scan index, *Liver mets* liver metastasis

The normal ranges of Hb, LDH and Alb were 11.3–14.5 g/dL, 116–199 IU/L and 4.2–5.4 g/dL, respectively

We evaluated the discriminatory ability of our models by quantifying the c-index. The c-index in our model including the BSI was 0.769 in the prediction of OS after docetaxel therapy. We also analyzed the discriminatory ability of the model excluding BSI by quantifying the c-index. The c-index of the model excluding BSI was 0.758 for the prediction of OS. In predicting OS, the c-index of the model including the BSI was higher relative to the model excluding the BSI.

**Fig. 4:**
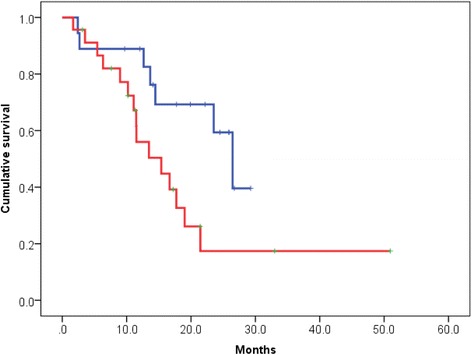
Kaplan–Meier curve for overall survival (OS) after docetaxel induction therapy according to the bone scan index (BSI). The blue line indicates survival for patients with a BSI ≤1 (*n* = 18) and the red line indicates survival for patients with a BSI >1 (*n* = 23). The median OS of patients with a BSI ≤1 and a BSI >1 was 26.5 and 15.4 months, respectively (*p* = 0.029)

We compared the survival probability by BSI categories. The median OS was 26.5 months in patients with a BSI ≤1 and 15.4 months in patients with a BSI >1 (*p* = 0.029). Patients with a BSI >1 had a significantly shorter OS than patients with BSI ≤1 (Fig. 4).

**Fig. 5:**
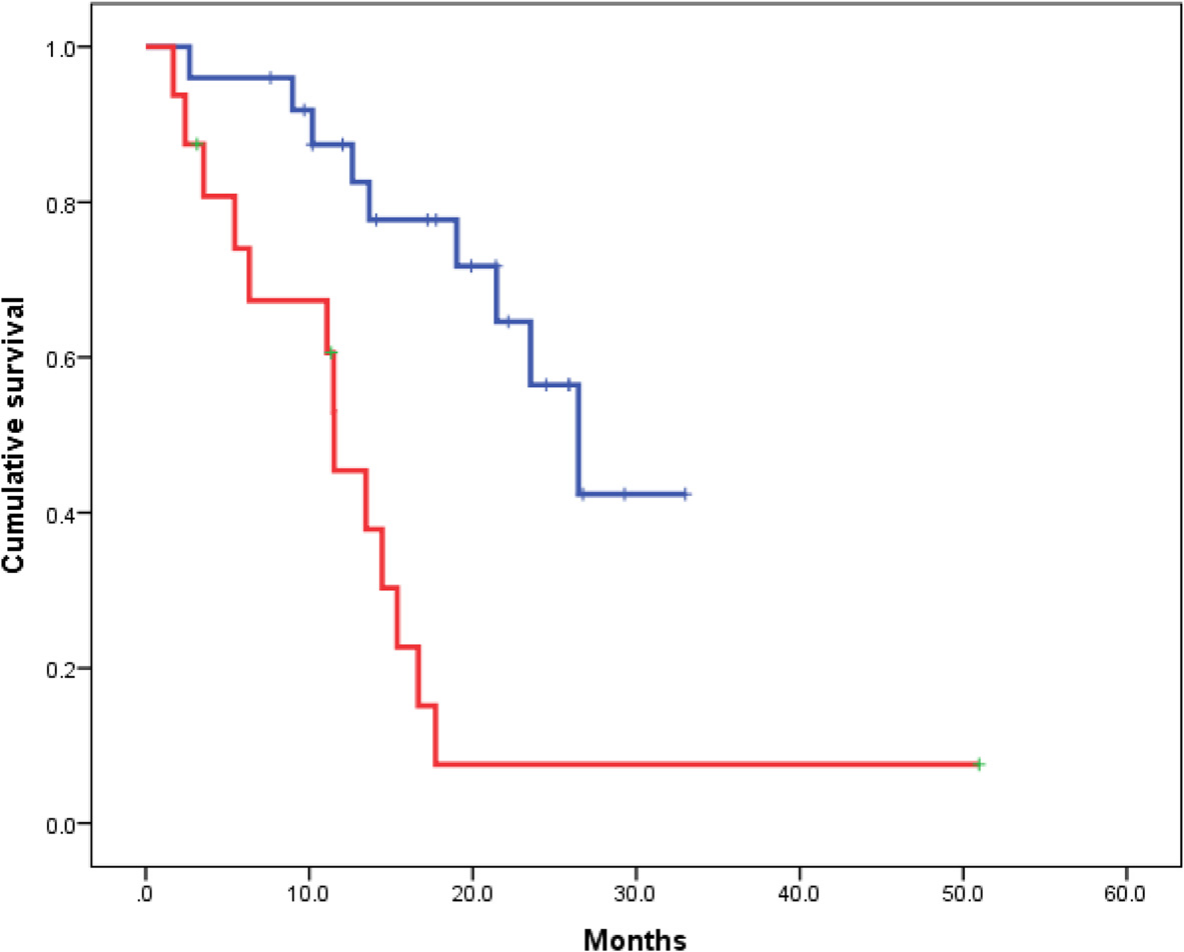
Kaplan–Meier curve for overall survival (OS) after docetaxel induction therapy according to risk group. We stratified the patients into two cohorts with low risk (0–1 risk factor present) and high risk (2–3 risk factors present). The blue line indicates survival for patients with low risk (*n* = 25) and the red line indicates survival for patients with high risk (*n* = 16). The median OS of patients with low risk and high risk was 26.5 and 11.5 months, respectively (*p* < 0.000). The risk factors in each risk group were shown in Table 3

We stratified the patients into two cohorts with low risk (0–1 risk factor present) and high risk (2–3 risk factors present). The risk factors in each risk group were shown in Table 3. We found a statistically significant difference in OS after docetaxel treatment among risk groups (Fig. 5; *p* < 0.000).

**Table 3:** Distribution of risk factors classified by risk group

	Low risk	High risk
Risk factors, n	0–1	2–3
Patients, *n* (%)	25 (61.0 %)	16 (39.0 %)
Alb, <3.9 g/dL, *n* (%)	6 (14.6 %)	14 (34.1 %)
BSI, >1 %, *n* (%)	9 (22.0 %)	14 (34.1 %)
Liver mets, yes, *n* (%)	1 (2.4 %)	5 (12.2 %)

There were no patients with >3 risk factors

*Alb* albumin, *BSI* bone scan index, *Liver mets* liver metastasis

## Discussion

In this study, we reported the usefulness of BSI which calculated by computer-aided diagnosis system involving bone scans as prognostic biomarker in CRPC patients, although the routine use of bone scan in CRPC treatments remains controversial. Recently, clinical significance of imaging in CPRC treatments have been increasing and developing rapidly. Several studies demonstrated the usefulness of the PET/CT both for restaging and for assessing the response to treatment in CRPC patients [27–29].

Several groups have reported prognostic models for the survival of patients with castration-resistant prostate cancer. Armstrong and colleagues reported that a decrease of 30 % in PSA level, visceral metastasis, anemia and bone scan progression were independent prognostic factors in docetaxel-treated patients with CRPC [20]. We have also previously described the risk factors for mCRPC patients before docetaxel treatment [18]. In results, pain, visceral metastasis, anemia and bone metastasis progression were independent prognostic factors for OS after docetaxel [18]. Because >80 % of mCRPC patients have bone metastases [4, 5], more attention should be given to accurate evaluation of bone metastases in the consideration of prognosis. The extent of disease (EOD) score suggested by Soloway et al. [30] was used for the evaluation of bone metastasis in prostate cancer. Based on the number or extent of the metastases, the scans were divided into the following five grades according to the EOD on bone scan [30]: 0) normal or abnormal as a result of benign bone disease; 1) <6 bony metastases, each of which is <50 % of the size of a vertebral body (one lesion that was approximately the size of a vertebral body was counted as two lesions); 2) 6–20 bone metastases, sized as described above; 3) >20 metastases but fewer than seen in a “superscan”; and 4) superscan or its equivalent (i.e., >75 % of the ribs, vertebrae and pelvic bones). Previously, we analyzed the relationship between prostate cancer outcomes and pretreatment clinical factors, and developed a prognostic survival model for patients with bone metastatic hormone-naive prostate cancer [31]. In that model, the EOD score was a strong prognostic factor for survival.

Although the EOD score has important prognostic information, it is a subjective and semi-quantitative parameter. For a more accurate and convenient method for bone metastases evaluation, objective and quantitative markers are required. Accurate evaluation of bone metastases would lead to an appropriate prediction of prostate cancer survival probability, and would be valuable for patient counseling.

It is anticipated that a BSI that uses a computer-aided diagnosis system for bone scans will become an objective and quantitative clinical tool for evaluating bone metastatic prostate cancer. The BSI has been reported as being useful as a survival predictor among men with prostate cancer with various conditions such as hormone-naïve prostate cancer or CRPC [22, 32–38].

In patients with CRPC, the usefulness of the BSI as a prognostic marker has been reported. Mitsui et al. found that patients with a decreased BSI after taxane-based chemotherapy had significantly longer OS than other patients [22]. They also reported that patients with a BSI ≥3.0 had reduced survival relative to men with a BSI <3.0 in the same cohort. Koboteh et al. also reported that patients with a decreased BSI after docetaxel had a better prognosis than other patients [35]. Dennis et al. reported that a change in BSI from baseline to 3–6 months after treatment for CRPC may provide prognostic information [33]. Armstrong et al. reported that the BSI, and change in the BSI over time, were independently associated with OS in patients with mCRPC in a tasquinimod treatment trial [32]. Although some studies have demonstrated the usefulness of the BSI, unfortunately few have included significant clinical information such as blood data and visceral metastasis. In previous studies, patient age and blood data such as serum Hb, alkaline phosphatase, LDH and CRP levels have been reported as predictive factors for patients with prostate cancer [39–41]. In our study, we analyzed the correlations between survival and clinical factors including variables at docetaxel induction therapy (PSA, patient age, liver metastasis, local therapy, Hb, LDH, Alb, PSA doubling time, and BSI) using the Cox proportional hazards regression model. EOD classifications and serum ALP level were significantly associated with the BSI value; EOD and serum ALP level were not included in the multivariate analysis because of multicollinearity. Three factors were identified as independent prognostic biomarkers for OS after docetaxel therapy as follows: liver metastases, Alb and BSI. Because the BSI has continuous covariations, it is better suited and accurate in the prediction of individual patient prognosis relative to EOD classifications.

In addition, we evaluated the discriminatory ability of our models including or excluding the BSI by quantifying the c-index. The BSI improved the c-index from 0.758 to 0.769 in predicting OS after docetaxel therapy. Median OS after docetaxel was 26.5 months in patients with a BSI ≤1 and the median OS after docetaxel was 15.4 months in patients with a BSI >1 (*p* = 0.029). Patients with a BSI >1 had a significantly shorter OS than patients with a BSI ≤1.

Recently, there has been rapid development in the treatment of CRPC. In the United States and other Western countries, some new effective agents for CRPC have been approved, including docetaxel, cabazitaxel, radium-223 dichloride, sipuleucel-T, abiraterone and enzalutamide [4, 42–47]. Unfortunately, treatment for CRPC in Japan was very limited until 2013 (cabazitaxel, abiraterone and enzalutamide were approved in 2014), even though docetaxel was approved in 2008 [48]. Thus, in our study, none of the patients received cabazitaxel, sipuleucel-T, abiraterone or enzalutamide. These agents could improve the survival of patients with CRPC, and our model could underestimate the prognosis when these new agents were used for CRPC.

Thus, the first limitation of our study is that our models were developed using data from the “docetaxel era.” For more accurate prediction of prognosis in patients with prostate cancer in the “post-docetaxel era,” more recently collected data are needed. The second limitation is the fact that patients enrolled had various health statuses and complications. Our models considered neither health status nor patient complications that may influence prostate cancer treatment outcomes [49–51]. Patients with prostate cancer are much older than those with other malignancies. Health status and complications should be classified in the rating score and included as predictive factors in the prognostic model. Bone pain at diagnosis is also a strong predictor of survival [51]. Unfortunately, data regarding pain at baseline were not available in this study. Moreover, no information was available on the number of patients received bisphosphonate or denosumab after the development of CRPC. Bisphosphonate or denosumab showed significant effect for reducing the skeletal-related events and improving progression-free survival although these agents except clodronate has not been shown to improve overall survival in randomized phase 3 controlled trial [52, 53]. Finally, our population was small and observation periods were relatively short. Evaluation of larger patient populations and long observations are warranted to establish the usefulness of the BSI as a prognostic factor. Although some study limitations exist, the BSI before treatment might be useful as a prognostic biomarker for hormone-naïve bone metastatic prostate cancer.

## Conclusions

In conclusion, we evaluated the prognostic value of a computer-aided diagnosis system (Bonenavi) for bone scans in patients who received docetaxel treatment for mCRPC. The BSI (calculated using Bonenavi), Alb and liver metastases were independent prognostic factors for OS after docetaxel therapy. BSI might be a useful tool for risk stratification of patients with mCRPC undergoing docetaxel treatment.

## Abbreviations

iPSA: Initial prostate-specific antigen
GS: Gleason scores
PSADT: PSA doubling time
LDH: Lactate dehydrogenase
Hb: Hemoglobin
Alb: Albumin
BSI: Bone scan index
